# Supplementary material for: Proteasomal degradation of NOD2 by NLRP12 in monocytes promotes bacterial tolerance and colonization by enteropathogens
Source: Nat Commun. 2018 Dec 17;9:5338. doi: 10.1038/s41467-018-07750-5 (PMC6297353; doi:10.1038/s41467-018-07750-5)
Supplement: Supplementary file 1 — Supplementary Information [file 41467_2018_7750_MOESM1_ESM.pdf]

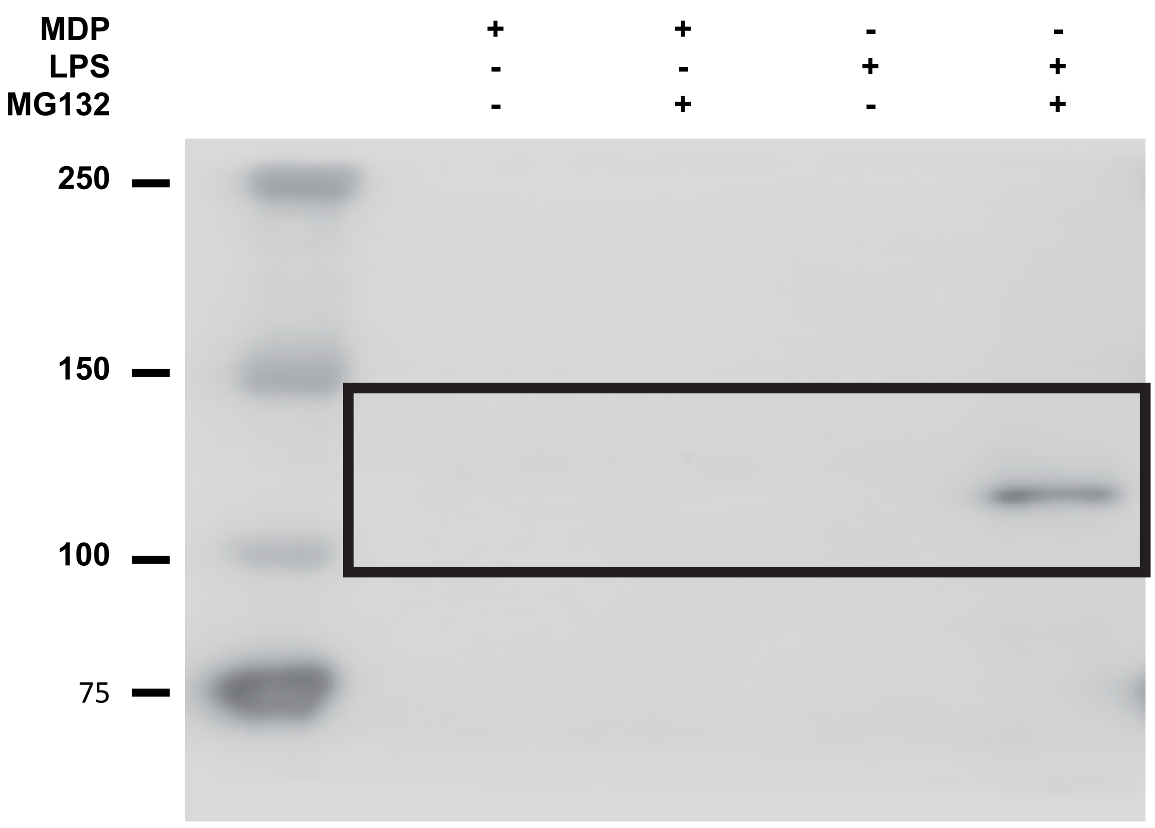

Supplementary Fig. 1. Complete membrane from Figure 1a (boxed area). Western blot of Co-IP protein Myc-NOD2. The protein was separated using a SDS-gel and transferred to PVDF membrane. The blot was incubated using an anti-NLRP12 (1:1000) antibody (GeneTex, Inc., USA).

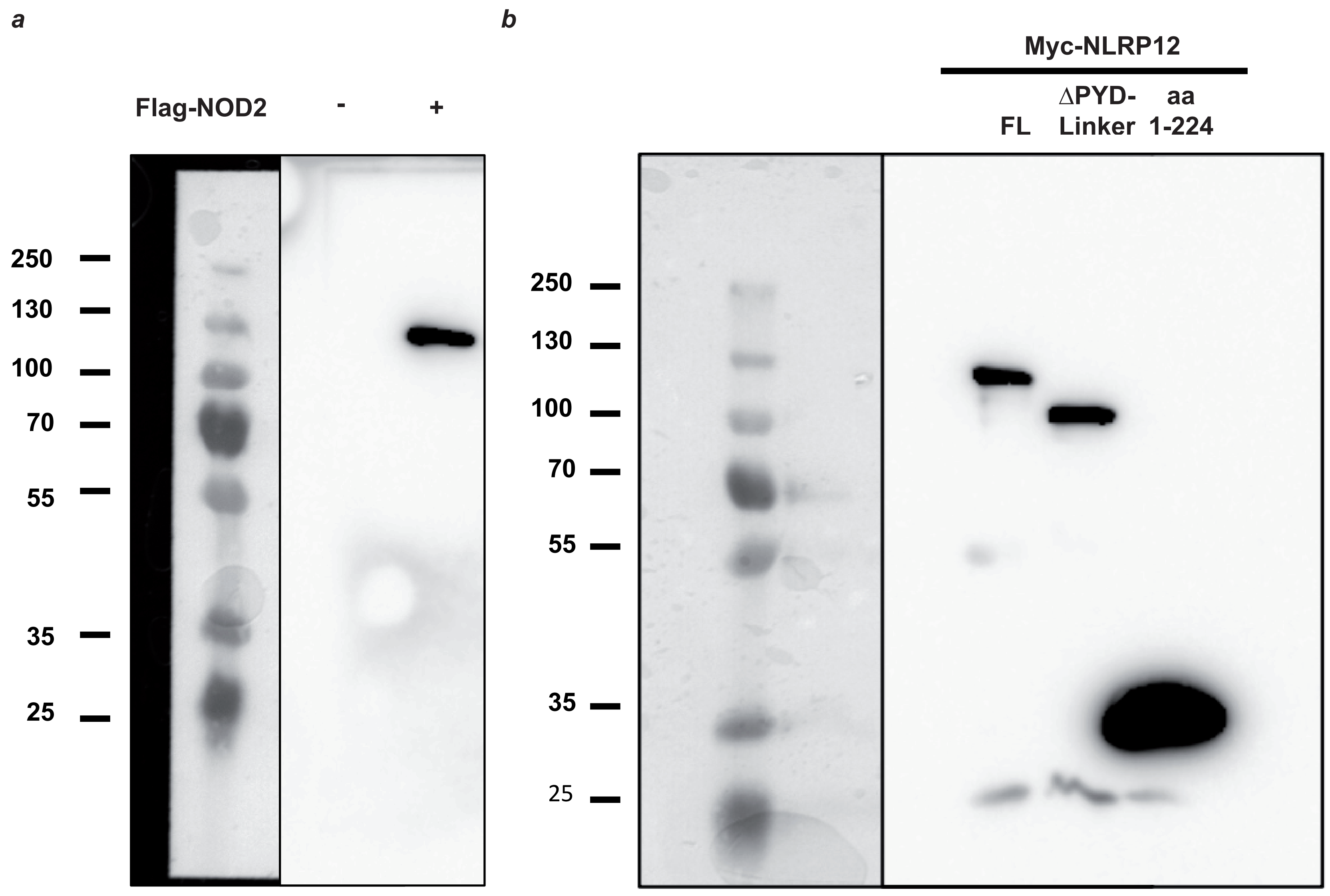

Supplementary Fig. 2. Representative Western blot from Figure 1d of ***a)*** NOD2 full-length and ***b)*** Myc-NLRP12 full-length (FL) and mutant isoforms. The protein was separated using a SDS-gel and transferred to PVDF membrane. The blot was incubated using a monoclonal anti-FLAG M2 (Stratagene, #200471) (1:2000).or a polyclonal anti-cMyc (Santa Cruz, sc-789) (1:1000) respectively

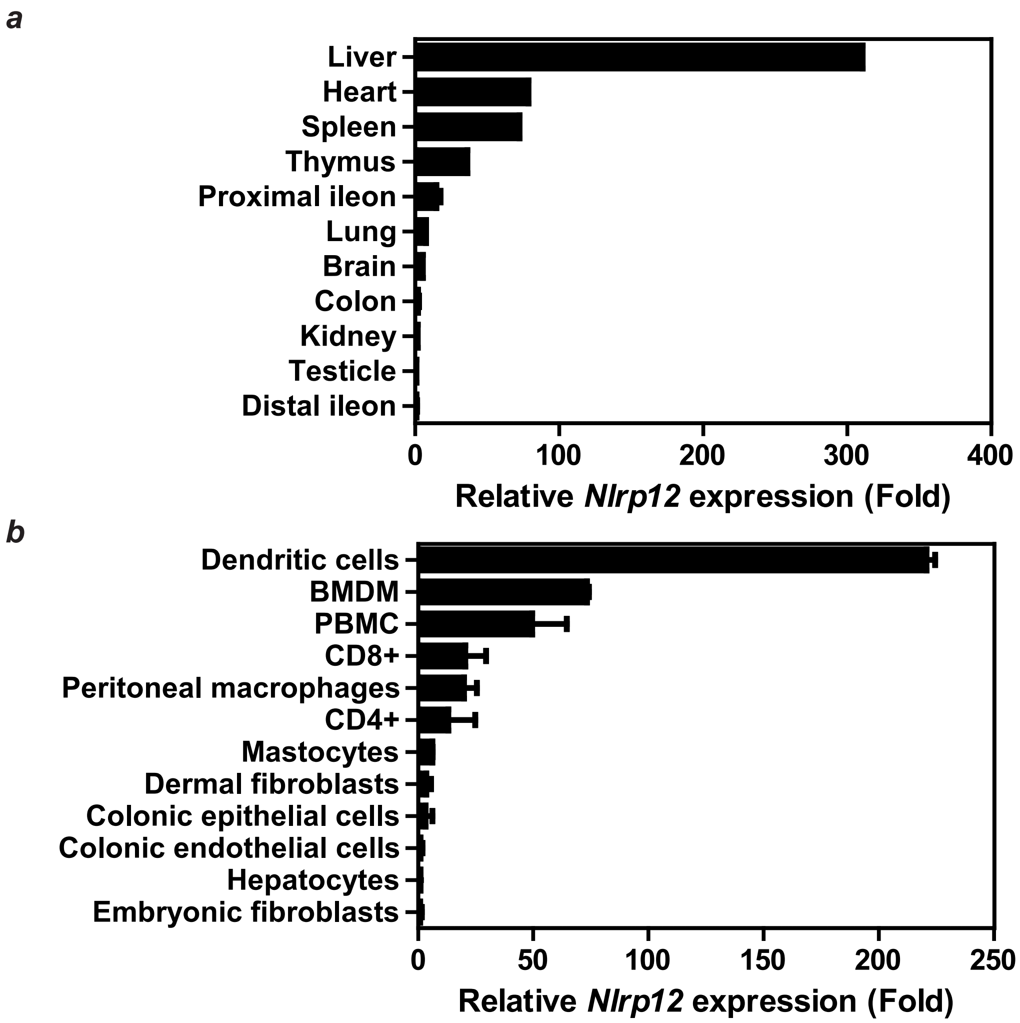

Supplementary Fig. 3. ***Nlrp12*** **gene expression pattern in mice by qRTPCR analysis.**

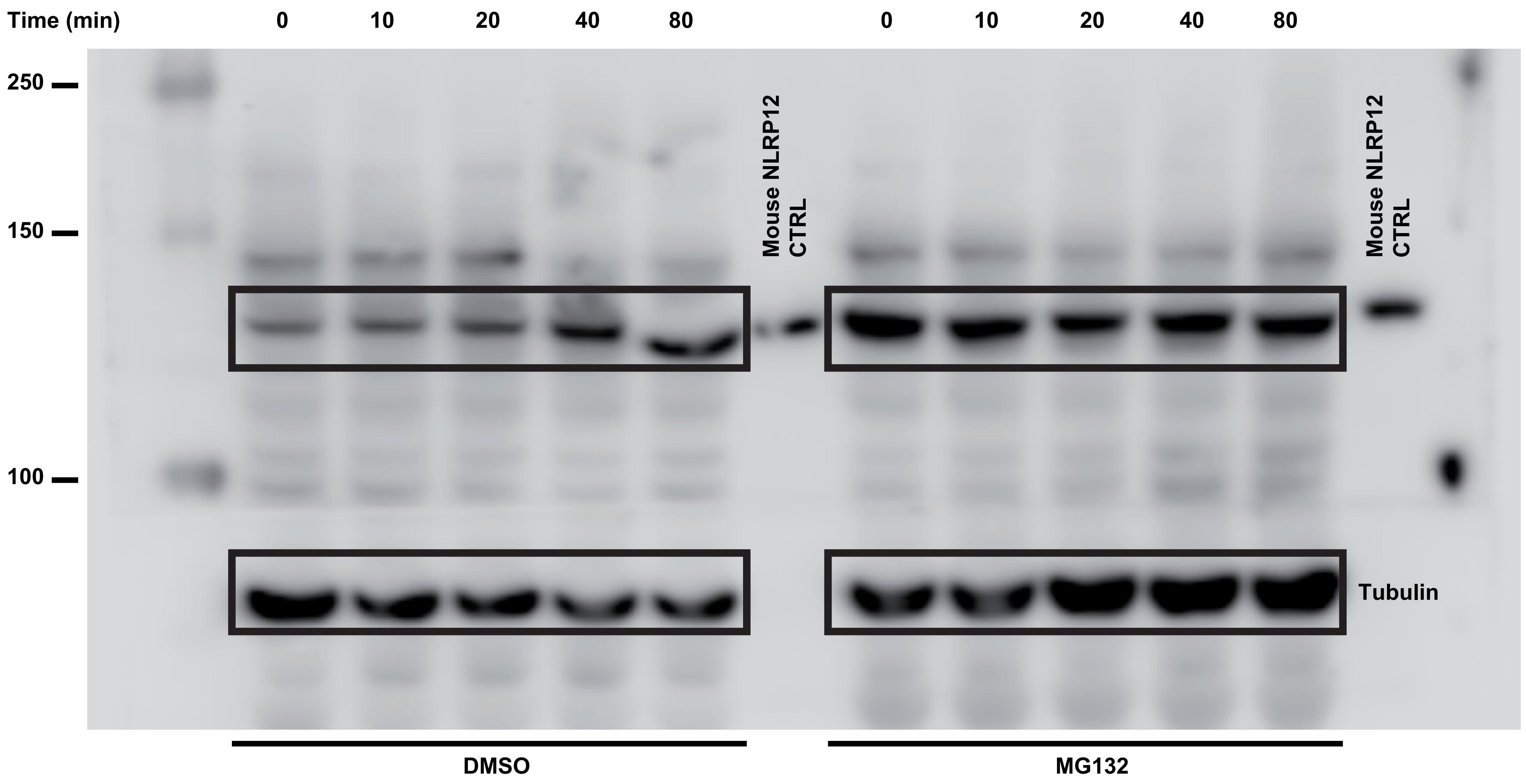

Supplementary Fig. 4. Complete membrane from Figure 2a (top band-boxed areas). Western blot of Co-IP protein Myc-NOD2. The protein was separated using a SDS-gel and transferred to PVDF membrane. The blot was incubated using a Monoclonal anti-pUb-K48 (Merck, Millipore, USA) (1:2500).

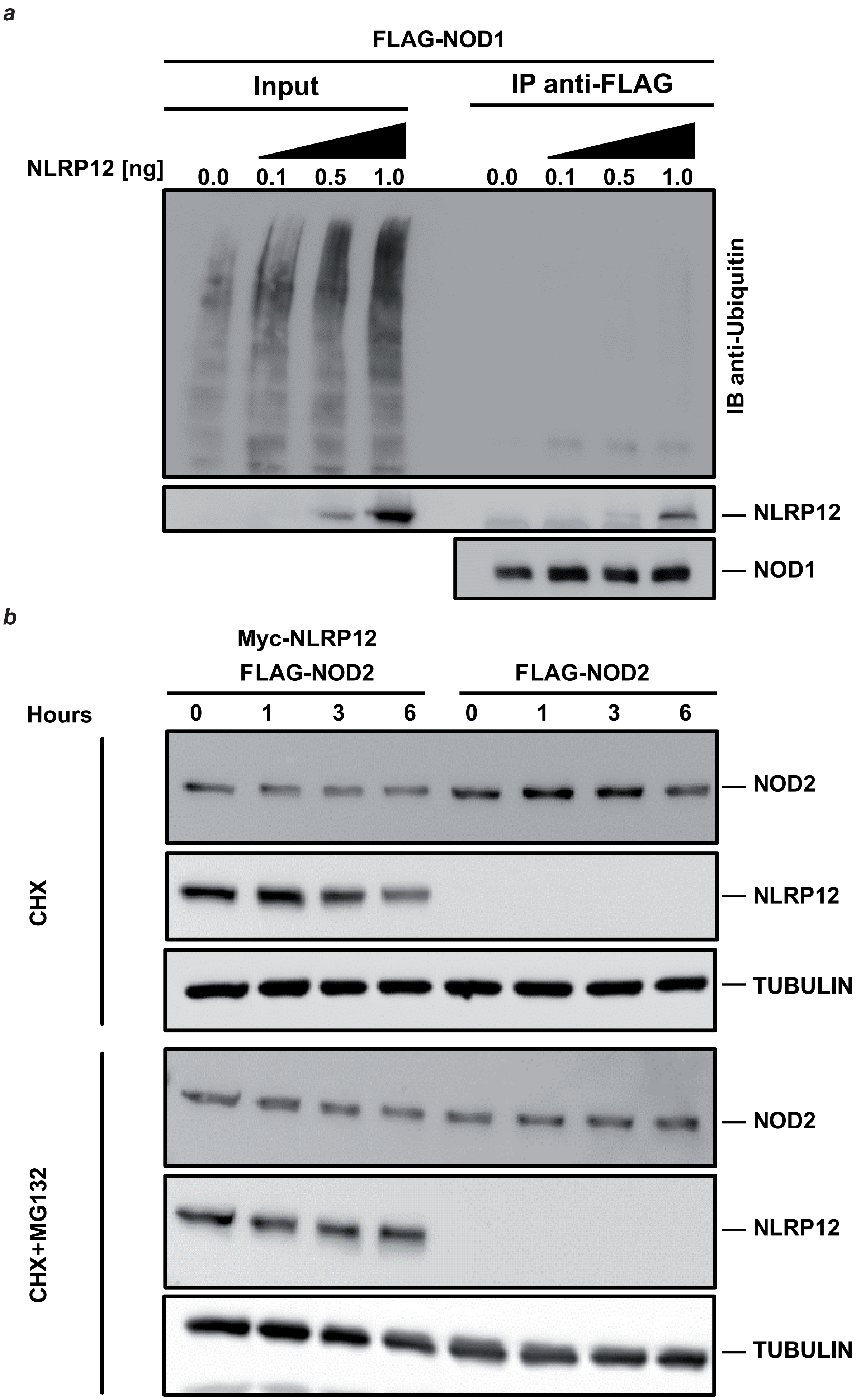

Supplementary Fig. 5. NLRP12 does not affect the stability of NOD1 protein. ***a)*** Western blot analysis of NOD1 ubiquitination in HEK293T cells that were transfected with FLAG-tagged NOD2 in the presence of increasing amounts of NLRP12. ***b)*** Western blot analysis of NOD2 stability in the presence of cycloheximide (CHX; 20 μg/ml) for 4 hour and of MG132 (12.5 μM). HEK293T Cell extracts were subjected to Western blot analysis for Flag-tagged NOD2, Myc-tagged NLRP12 and Tubulin.

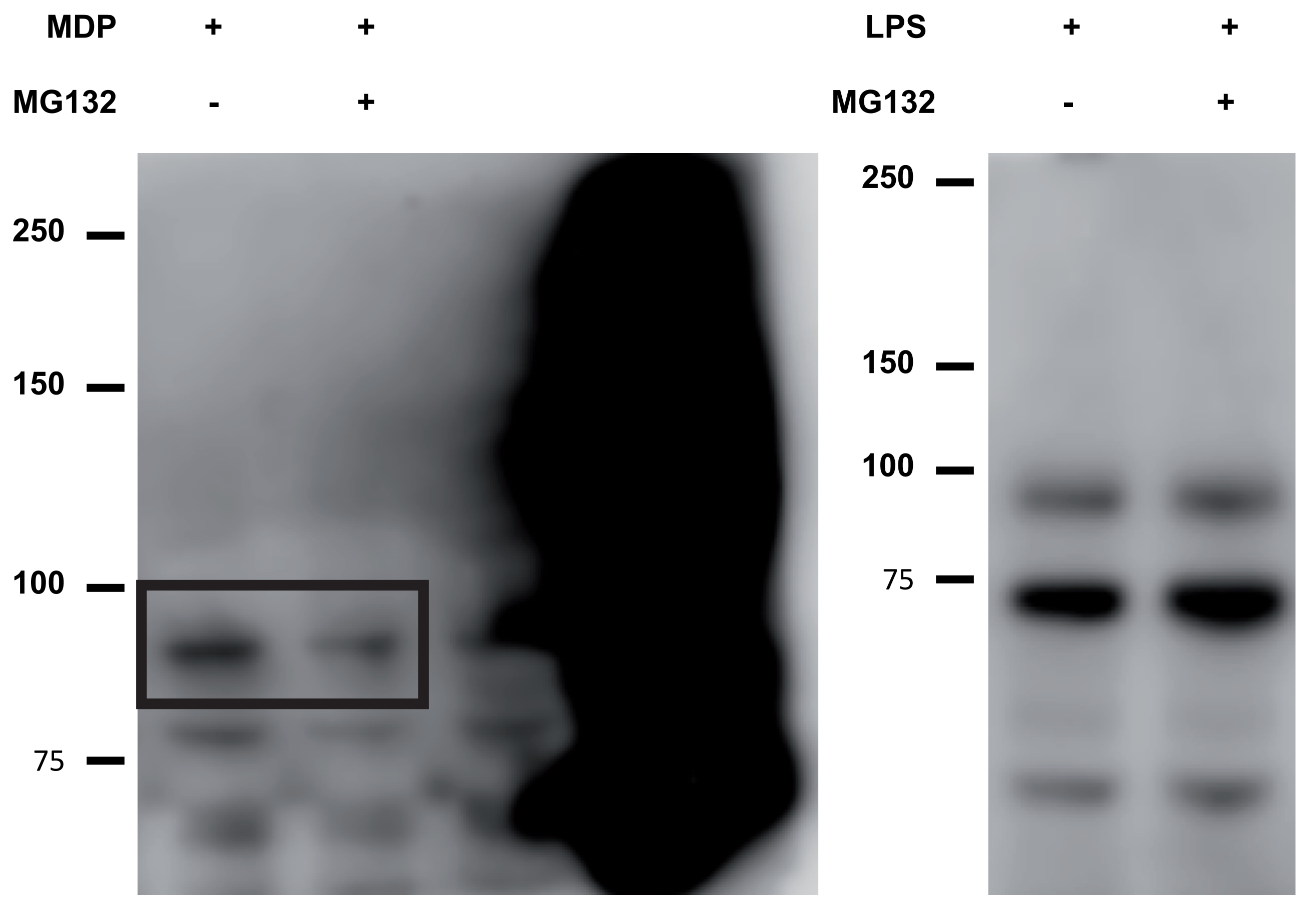

Supplementary Fig. 6. Complete membrane from Figure 2d (boxed areas). Western blot of Co-IP protein Myc-NOD2. The protein was separated using a SDS-gel and transferred to PVDF membrane. The blot was incubated using a monoclonal anti-HSP90alpha/beta (F8) (Santa Cruz Biotechnology Inc., USA) (1:1500).

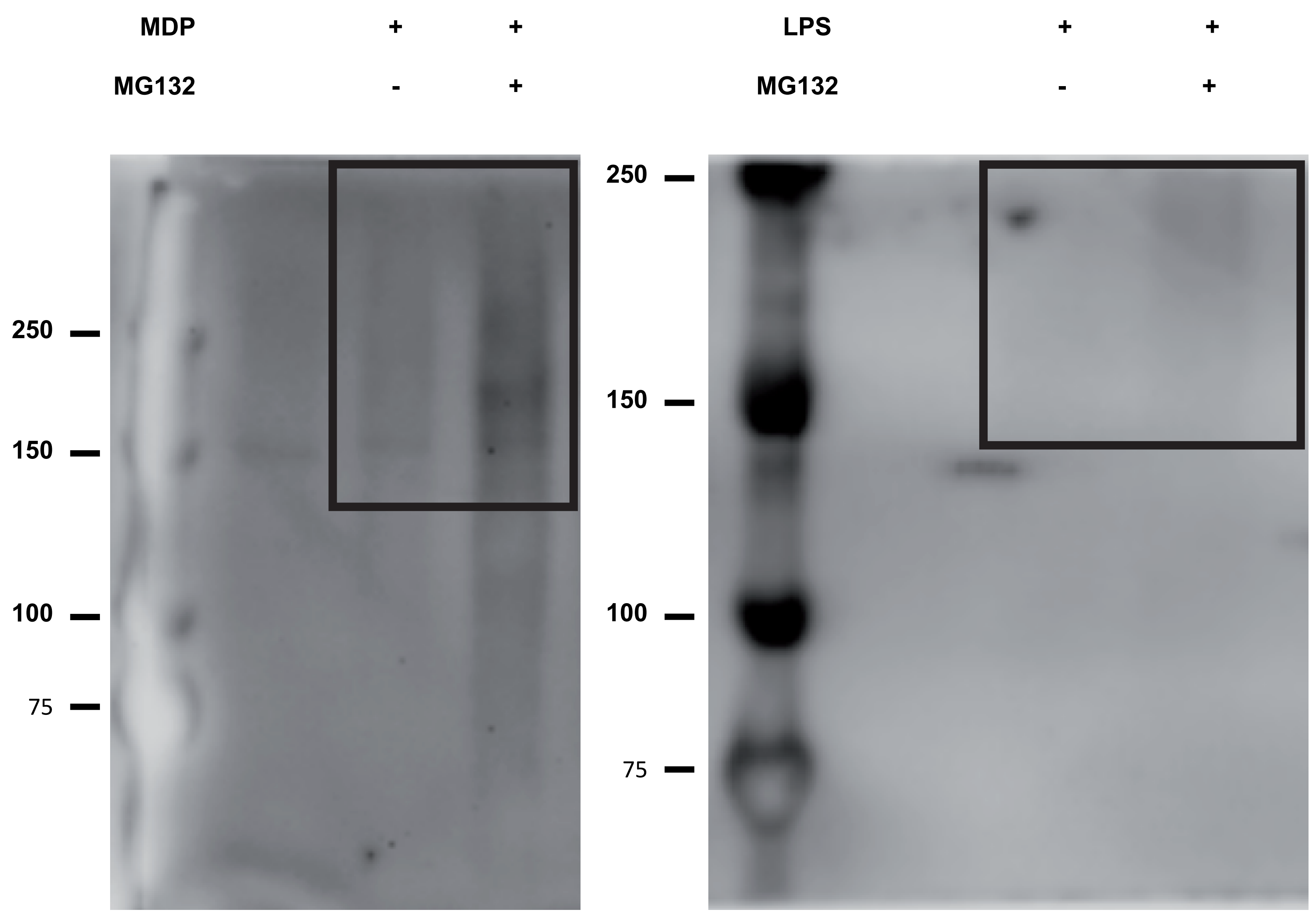

Supplementary Fig. 7. Complete membrane from Figure 2d (boxed areas). Western blot of Co-IP protein Myc-NOD2. The protein was separated using a SDS-gel and transferred to PVDF membrane. The blot was incubated using a Monoclonal anti-pUb-K48 (Merck, Millipore, USA) (1:2500)

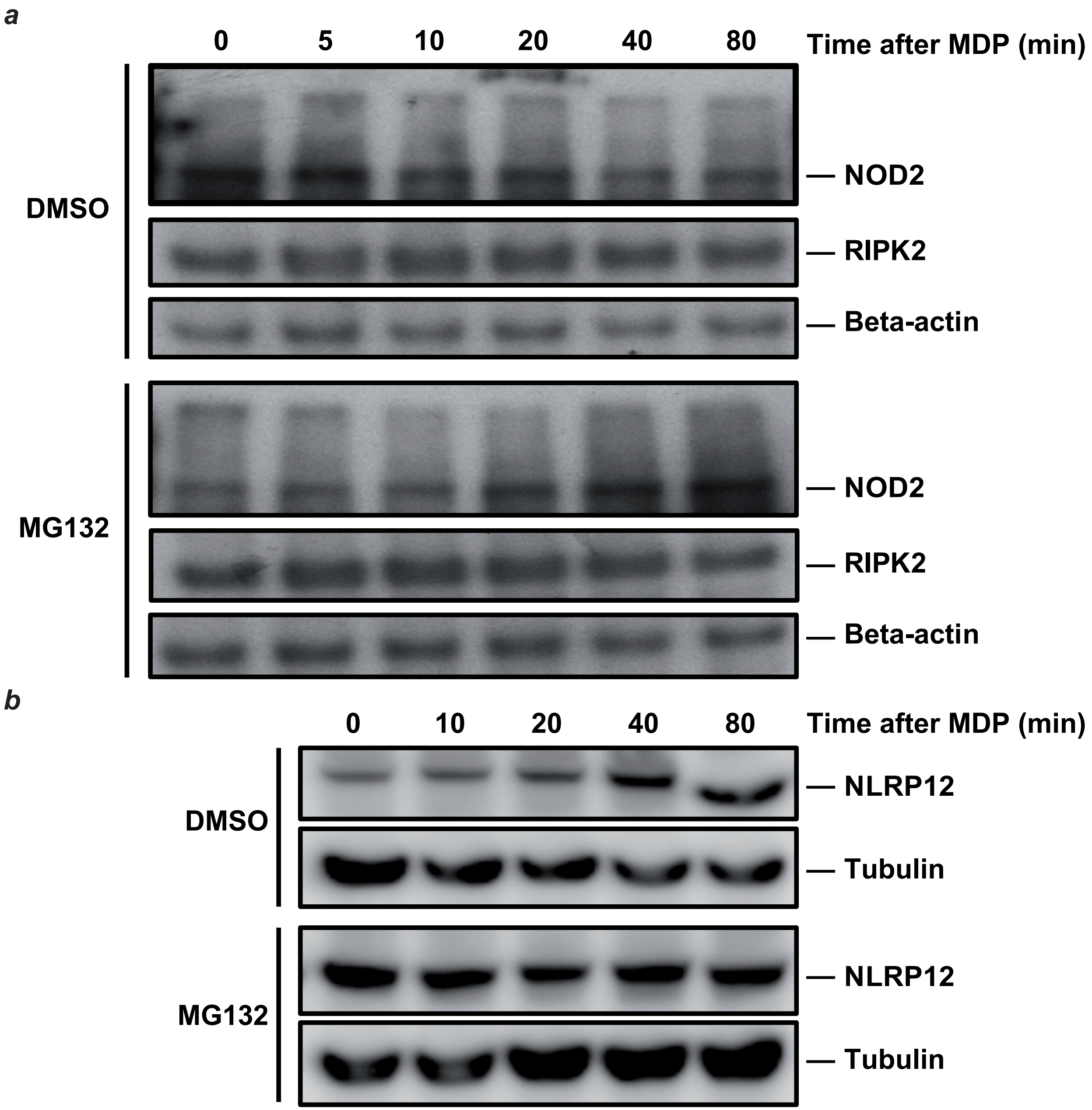

Supplementary Fig. 8. MDP enhances NOD2 degradation and NLRP12 expression. Cells were treated with DMSO or MG132 for 1h with DMSO or MG132. ***a)*** Immunoblot of extracts of THP-1 Myc-BirA*-NOD2 cells that treated or not with MDP at 10µg/mL (where indicated) and reaction stopped using RIPA buffer at the indicated time. Membranes were blotted against the NOD2-monoclonal antibody and RIPK2 and Actin as a controls. ***b)*** Immunoblot of extracts of THP-1 cells that were treated with MDP (10µg/mL) or LPS (0.2 µg/mL). Membranes were blotted against the NLRP12 antibody and Tubulin as a control.

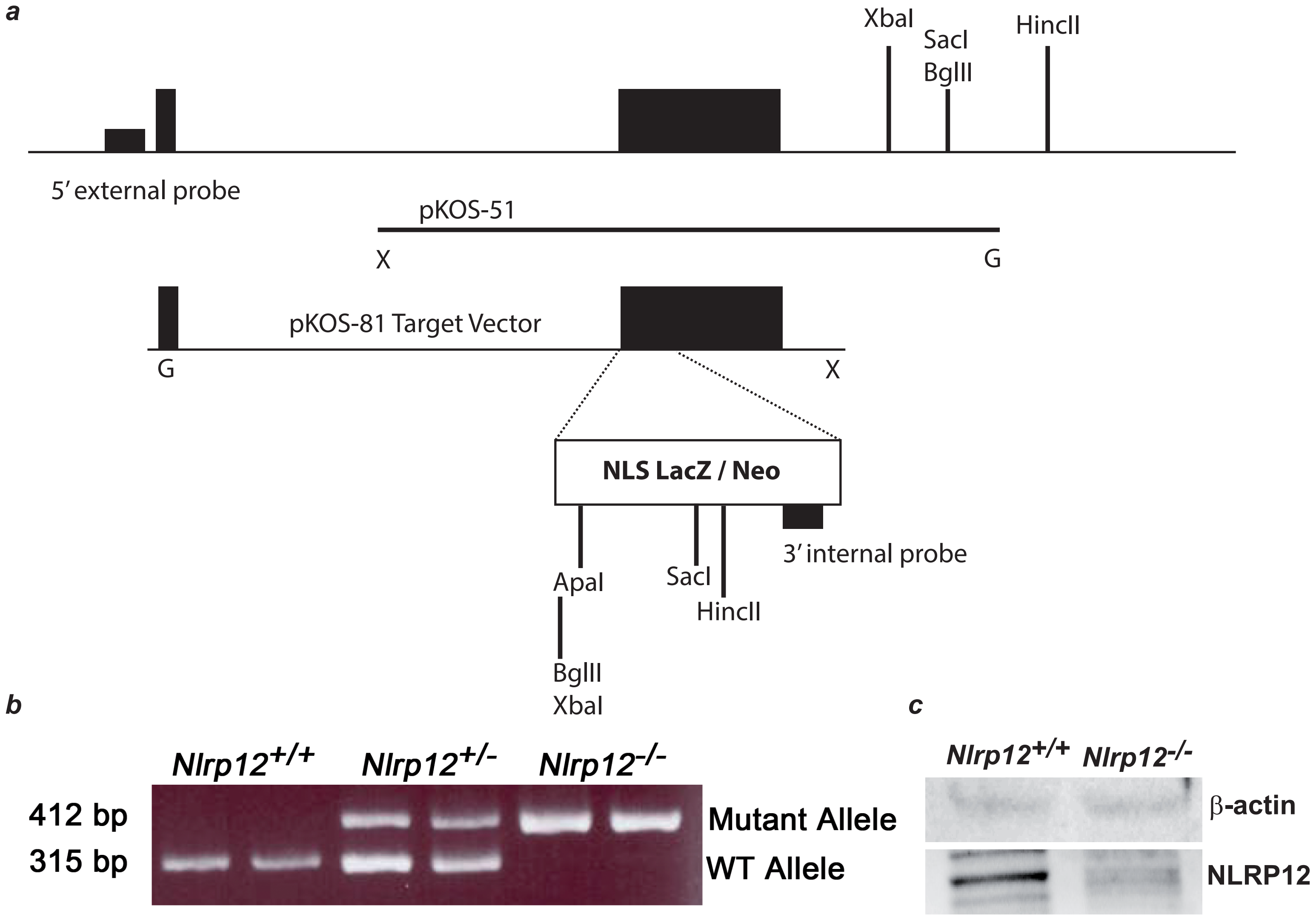

Supplementary Fig. 9. Generation of *Nlrp12*-deficient mice. ***a)*** Scheme of genetic ablation of NLRP12 expression in mice. ***b)*** Genotyping of *Nlrp12*-deficient mice by PCR on mouse tail genomic DNA. The bands corresponding to mutant and wild-type alleles are depicted. ***c)*** Western-blot analysis of NLRP12 expression in wild-type and *Nlrp12*-deficient mice.

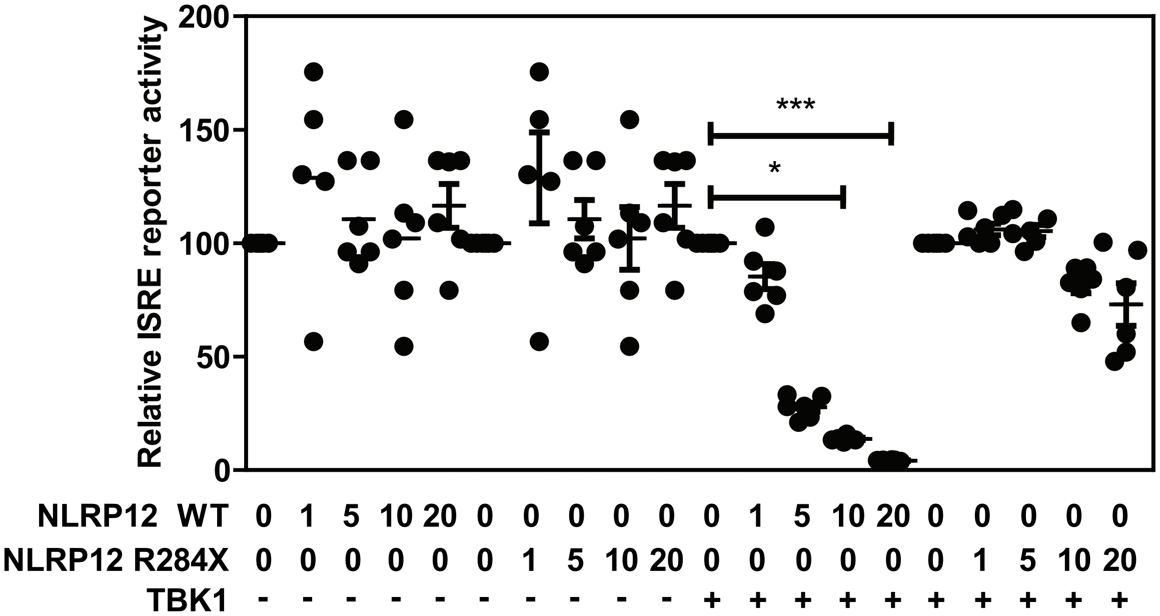

Supplementary Fig. 10. NLRP12 Negatively Regulates Type I IFN Signaling. ISRE-Luciferase activity in HEK-293T cells normalized to expression of β-galactosidase. Depicted are mean ± SEM (n=3).

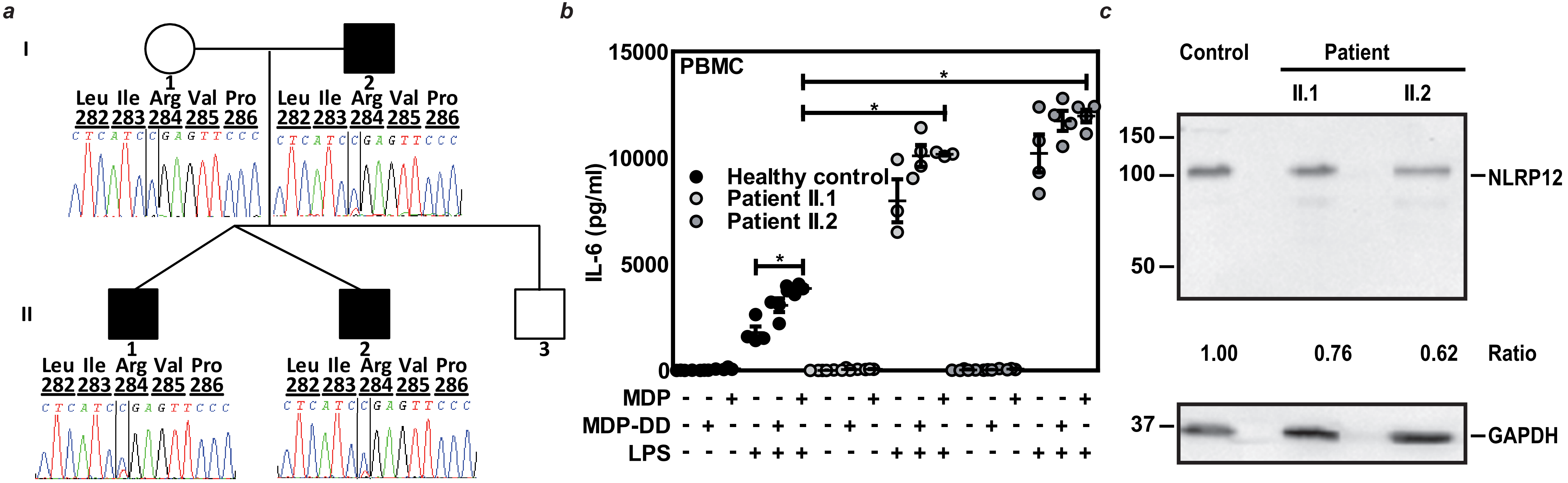

Supplementary Fig. 11. Loss of tolerance in PBMCs of FCAS2 patients is linked to a NLRP12 haploinsufficiency as a consequence of the activation of a surveillance pathway referred to as nonsense-mediated mRNA decay. ***a)*** Genealogical tree of a family with FCAS2 patients. Filled symbols represent patients with recurrent fever syndrome, and open symbols indicate unaffected family relatives. ***b)*** ELISA analysis of IL-6 secretion by PBMCs of healthy donors and twin patients carrying the nonsense R284X mutation in the *NLRP12* gene. ***c)*** Immunoblot analysis of NLRP12 expression in PBMCs of a healthy donor and both twin patients carrying the nonsense R284X mutation in the *NLRP12* gene.

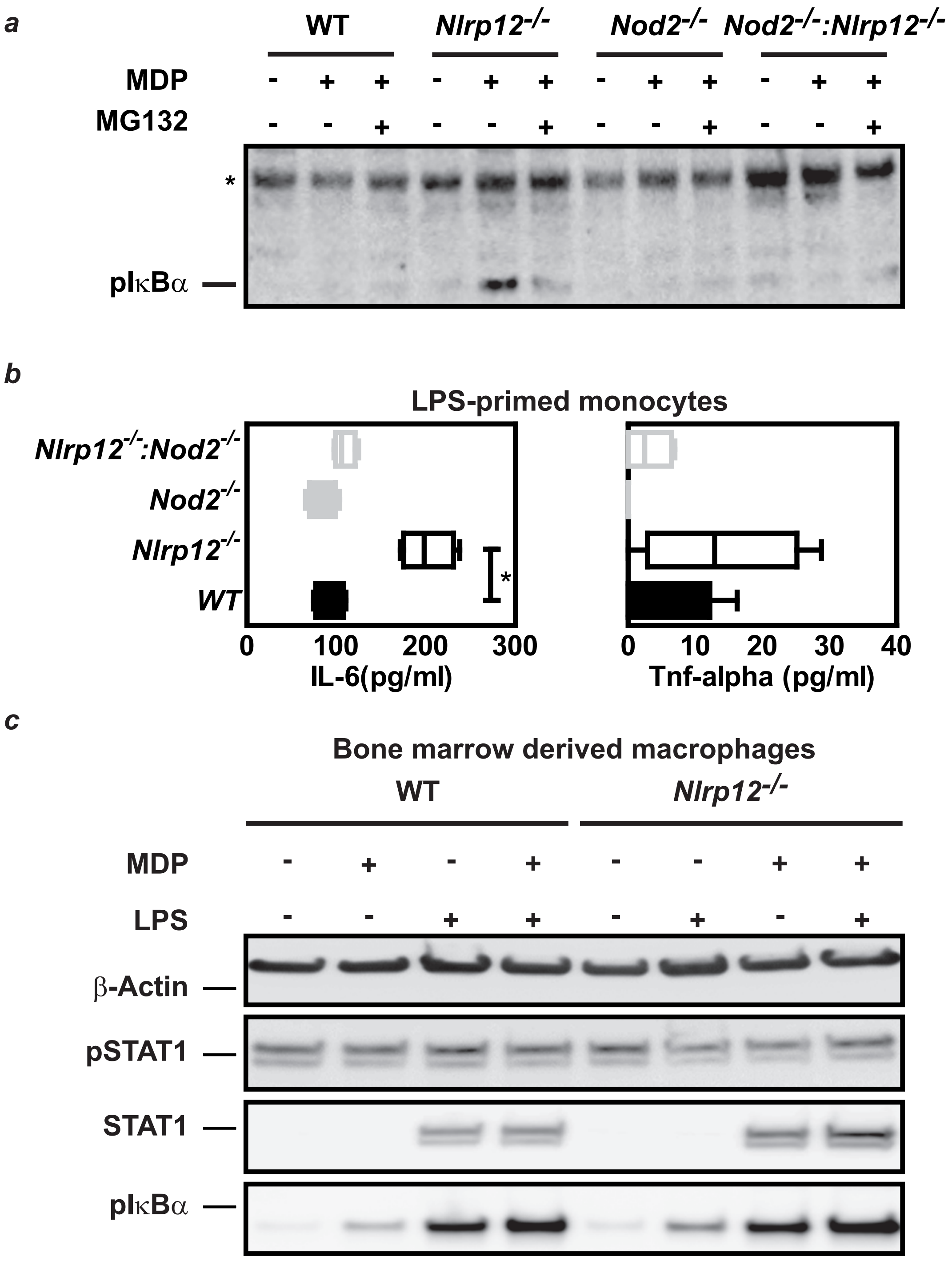

Supplementary Fig. 12. Loss of MDP tolerance in monocytes and macrophages from the bone marrow from *Nlrp12*-deficient mice as a consequence of enhanced NOD2 signaling. ***a)*** Immunoblot using anti-phospho IκB of extracts from monocytes that were treated by MDP and/or MG132 after being isolated from the bone marrow of mice that are deficient or not for NOD2 and/or NLRP12. The symbol * refers to non-specific band. ***b)*** MDP-induced secretion of TNF-alpha and IL-6 in the supernatant from monocytes derived from the bone marrow of control mice and of those that are deficient for NLRP12 and/or NOD2. The cells were were primed 4 hours with LPS before being incubated for 24 hours with MDP at 10µg/mL. All experiments were performed in quadruplicate. ***c)*** Immunoblot using anti-phospho IκB, anti-phospho STAT1 of extracts from differentiated macrophages that were treated by MDP and/or LPS.

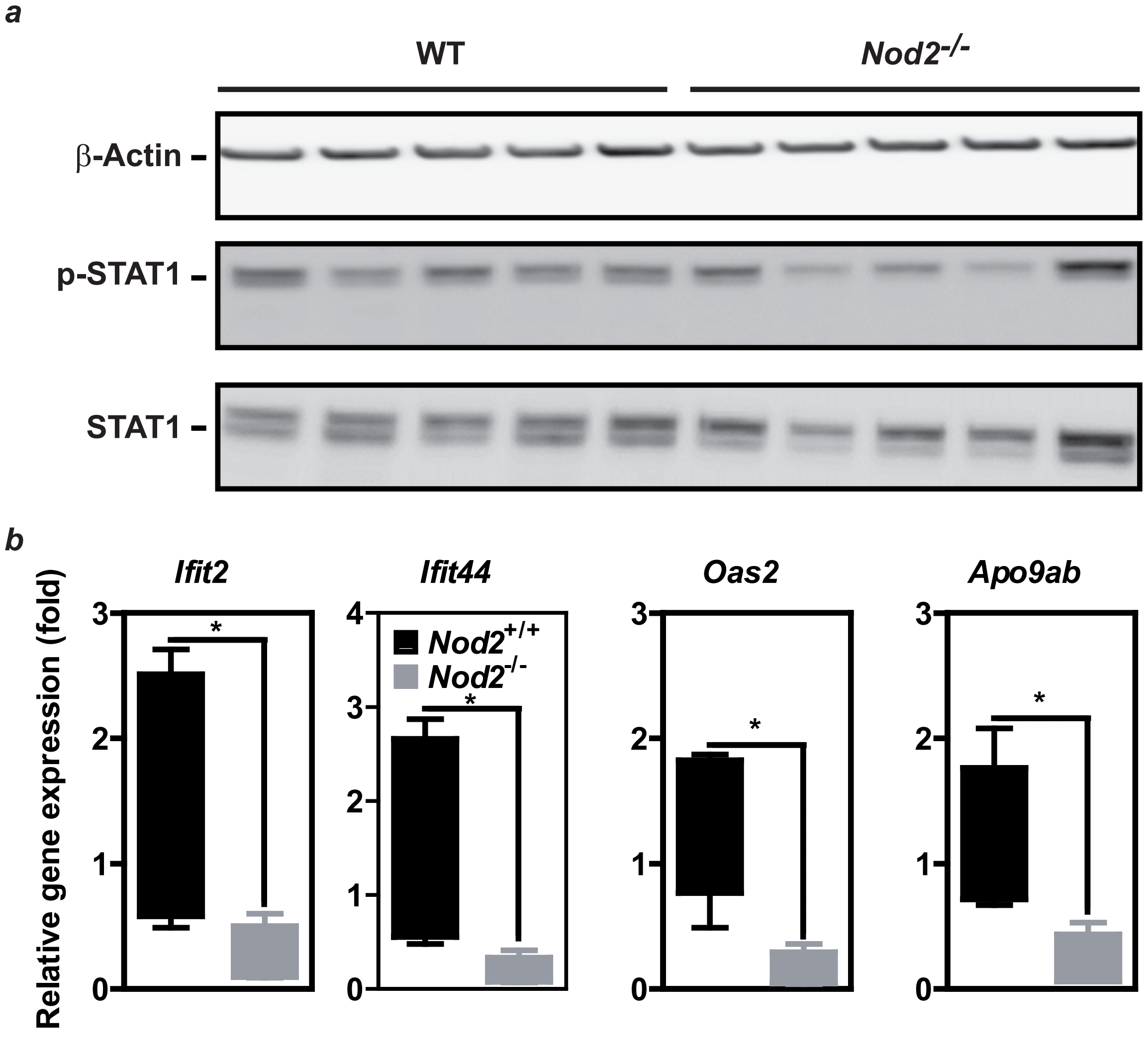

Supplementary Fig. 13. Loss of NOD2 impairs STAT3 activation and expression of STAT1-target genes in response to *Citrobacter rodentium*. *a)* Western blot analysis of STAT1 phosphorylation was performed by using total tissue samples from proximal colon of *Nod2*$^{-/-}$ mice and wild-type controls. *β-Actin* was used as loading control. ***b)*** Relative gene expression of *Ifit2*, *Ifit44*, *Oas2* and *Apo9ab* was analyzed at the proximal colon of *Nod2*$^{-/-}$ mice (n = 4) and controls (n = 5). Statistical significance was calculated by non-parametric Mann-Whitney test. * $P < 0.05$ and ** $P < 0.01$.

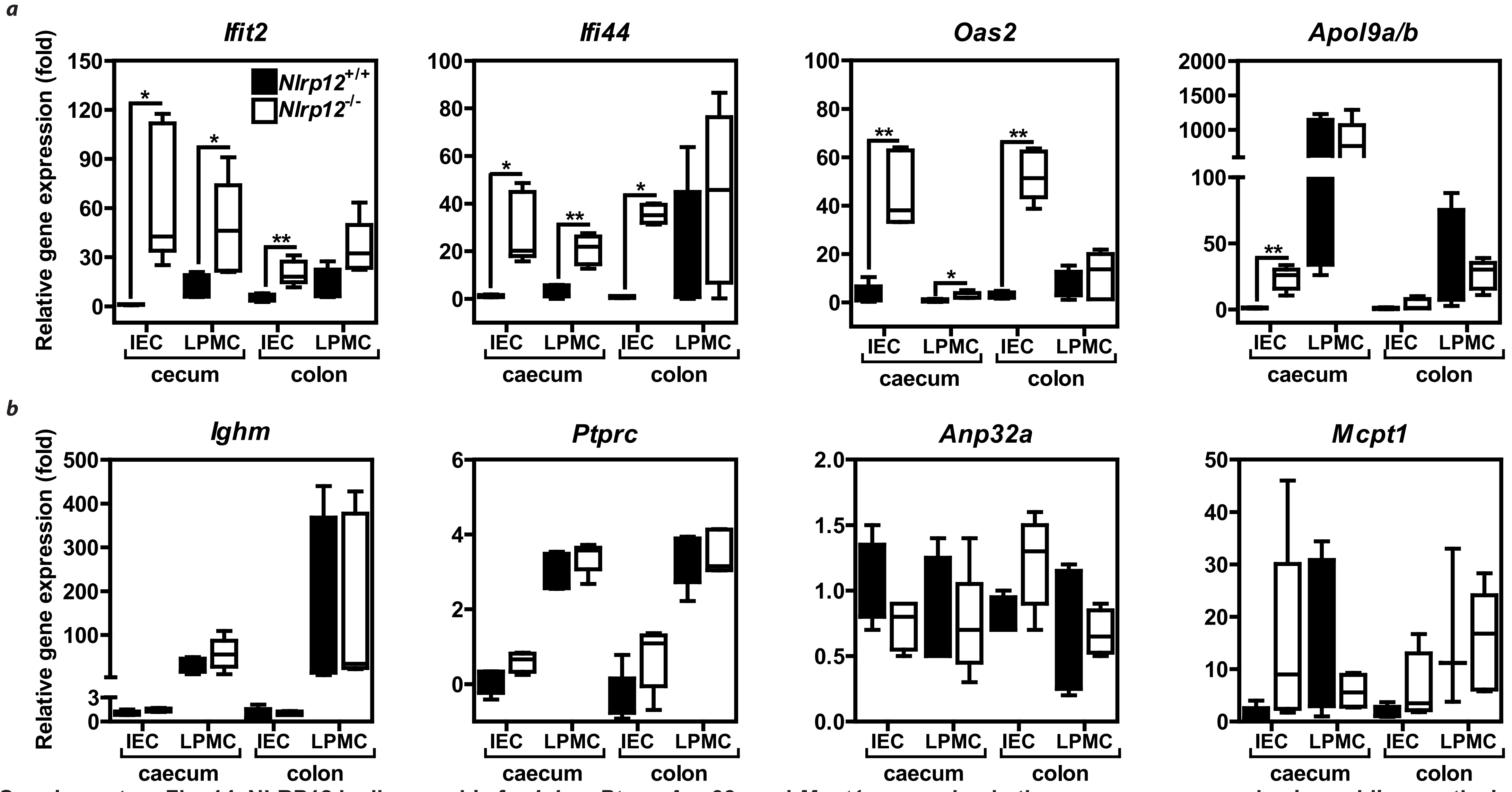

Supplementary Fig. 14. NLRP12 is dispensable for *Ighm, Ptprc, Anp32a* and *Mcpt1* expression in the mouse caecum and colon, while negatively regulating the expression of several ISGs. ***a)*** Relative gene expression of *Ifit2, Ifit44, Oas2* and *Apol9ab* was analyzed by qRT-PCR using IEC and LPMC isolates (n = 5) of caecum and proximal colon from *Nlrp12*$^{-/-}$ mice and wild-type controls. ***b)*** Relative gene expression of *Ighm, Ptprc, Anp32a,* and *Mcpt1* was analyzed by using IEC and LPMC isolates (n = 5) of caecum and colon from *Nlrp12*$^{-/-}$ and wild-type mice. Statistical significance was calculated by non-parametric Mann-Whitney test.

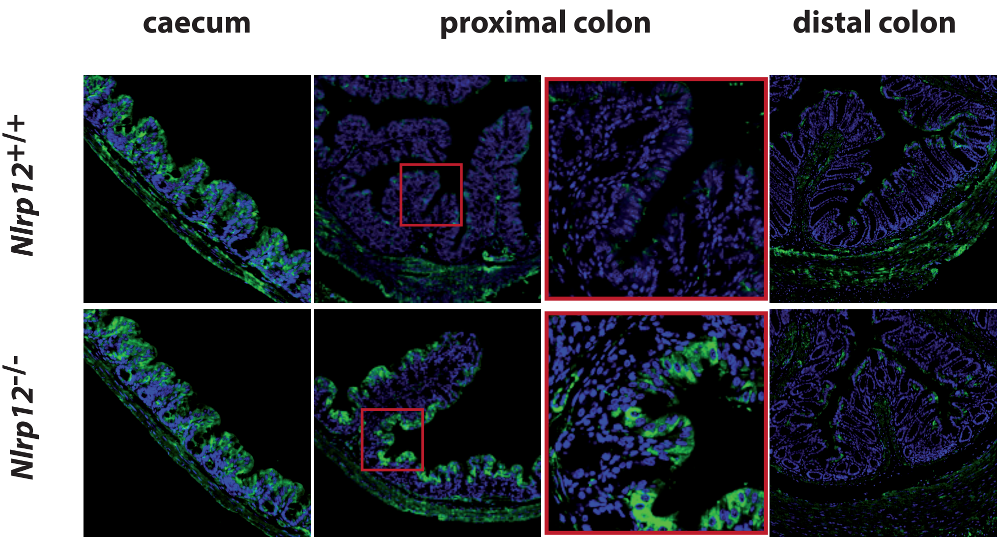

Supplementary Fig. 15. Loss of NLRP12 triggers OAS2 expression within the colonic epithelium. Immunohistochemistry for OAS2 was performed on 5 µm-thick tissue sections from caecum, proximal and distal colon of naïve wild-type and *Nlrp12*$^{-/-}$ mice. Scale bars represent 50µm (caecum), 200µm (proximal colon) and 100µm (distal colon).

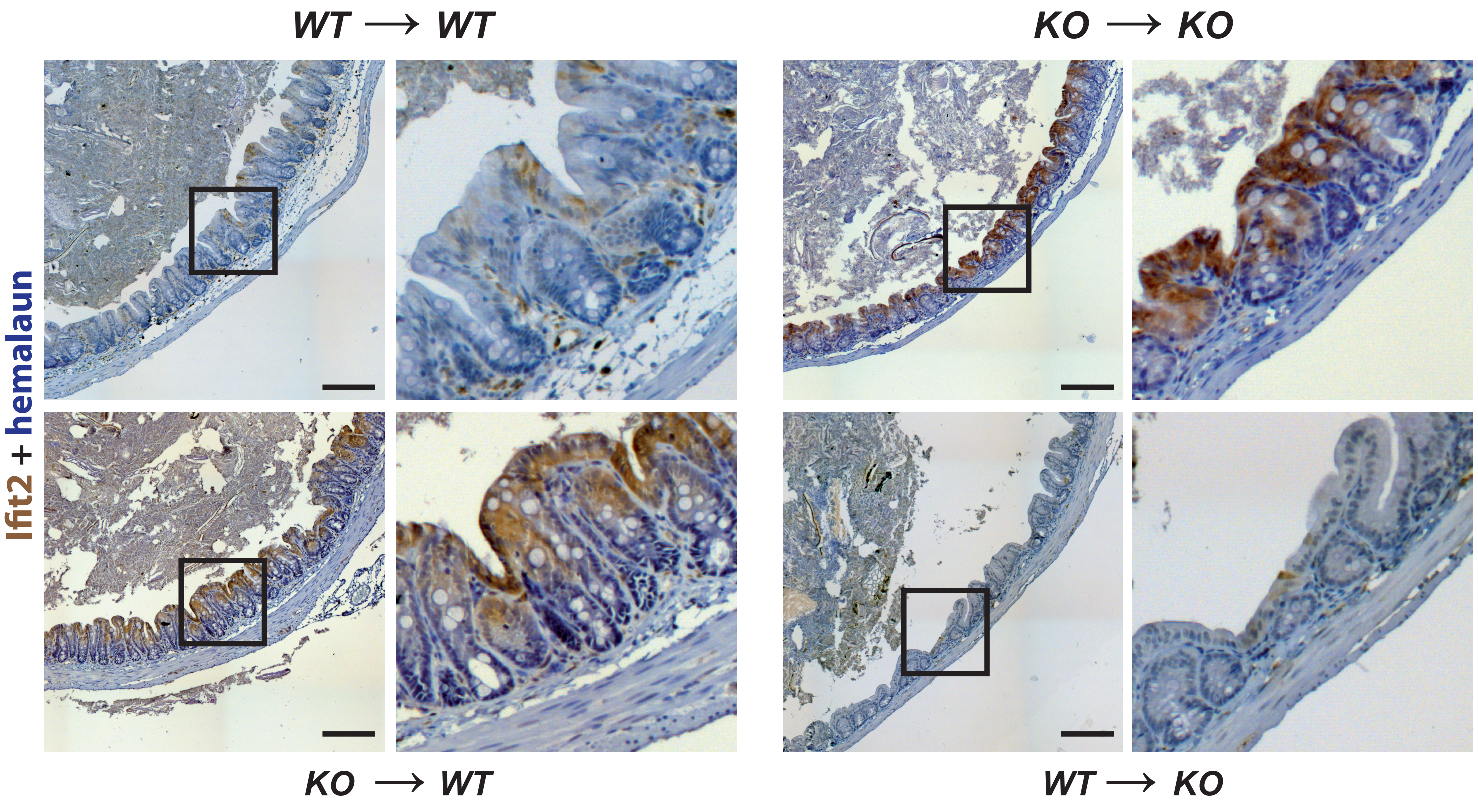

Supplementary Fig. 16. Loss of NLRP12 in leukocytes results in accumulation of inflammatory monocytes that triggers ISG induction within the colonic epithelium. IFIT2 protein expression was analyzed by immunohistochemistry on tissue sections prepared from caecum of wild-type chimeric mice (WT→WT), *Nlrp12*-deficient recipients that were reconstituted with hematopoietic cells from mutant mice (*Nlrp12*$^{-/-}$→N*lrp12*$^{-/-)}$, wild-type recipients that were reconstituted with hematopoietic cells from *Nlrp12*-deficient mice (N*Nlrp12*$^{-/-}$→WT), and knock-out mice that were reconstituted with hematopoietic cells from *Nlrp12*-deficient mice (WT→*Nlrp12*$^{-/-}$). Scale bars represent 200μm.

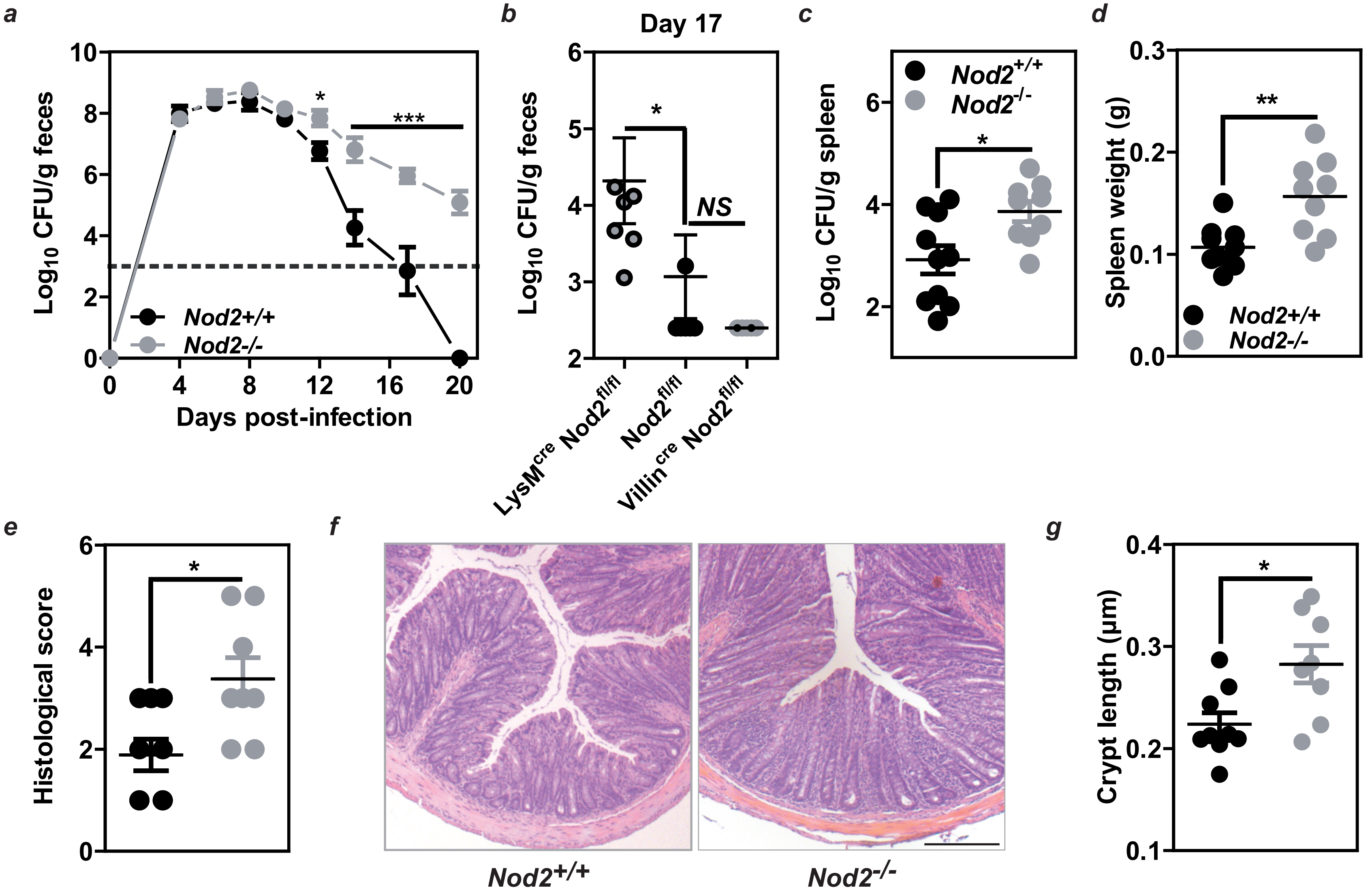

Supplementary Fig. 17. Loss of NOD2 in mononuclear cells impairs colonization resistance towards *Citrobacter rodentium*. ***a)*** Whole infection course in control and *Nod*2-deficient mice was monitored by quantifying colony-forming units (CFU) per gram feces. Detection limit of CFU assay is indicated by the dotted line. ***b)*** *C. rodentium* burden in faeces from either LysMCre;*Nod2*$^{fl/fl}$ (n=8), VillinCre;*Nod2*$^{fl/fl}$ (n=5) or *Nod2*$^{fl/fl}$ (n=8) mice at day 17 post-infection. Data represent mean ± SEM. **c)** to **g)** Susceptibility to *C. rodentium* infection in *Nod2*-deficient mice was assessed on day 20 post infection by quantifying **c)** CFU from spleens, **d)** spleen weights and **e)** histological scores. **f)** Representative H&E staining of 5 µm-thick tissue sections from distal colon and **g)** crypt lengths from distal colon.

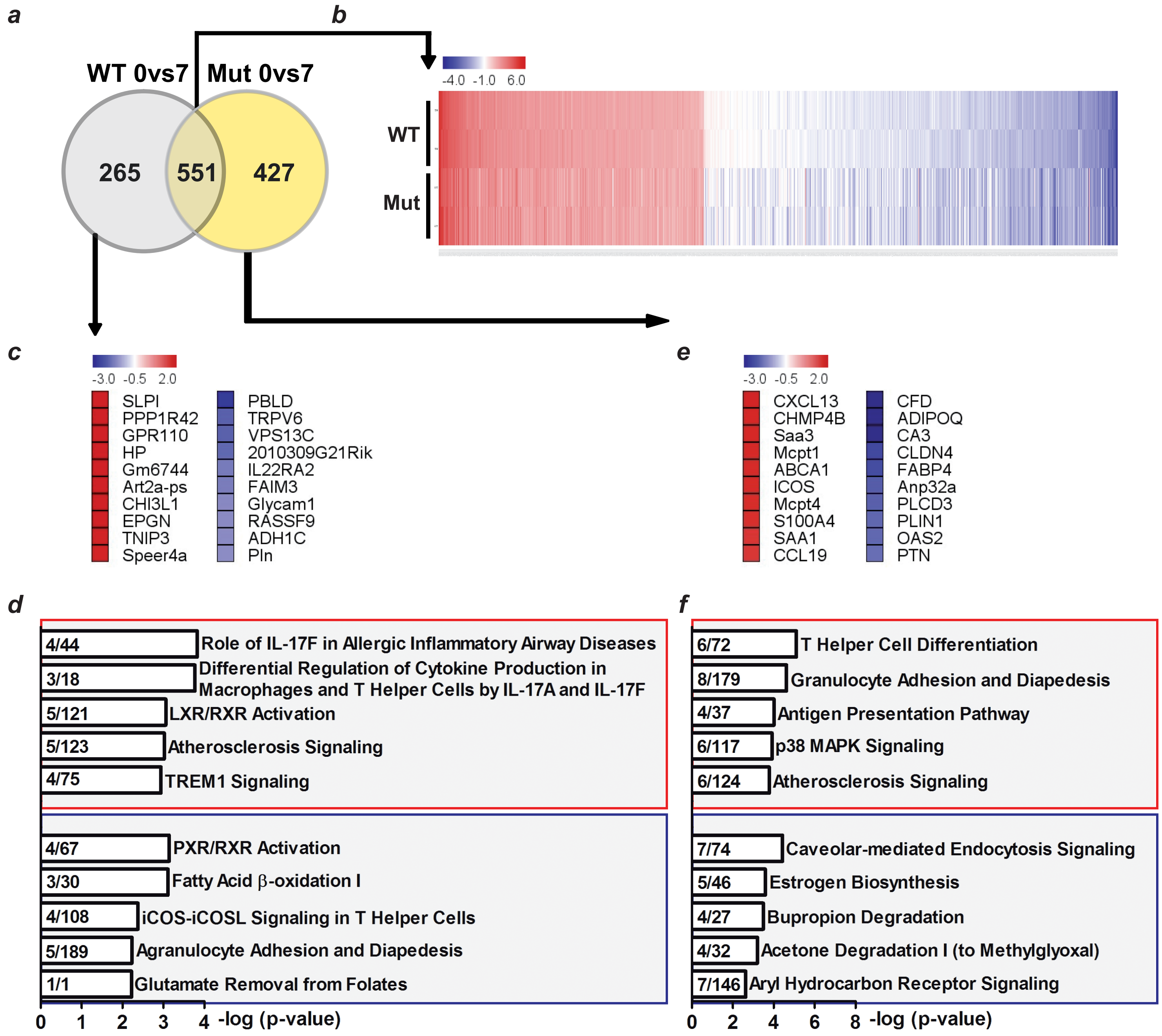

Supplementary Fig. 18. *Citrobacter rodentium* induces a distinct transcriptional response in *Nlrp12*-deficient mice. *a)* Venn diagram analysis of differentially expressed genes between infected *lrp12*$^{-/-}$ and wild-type mice at day 7 post infection. ***b)*** Heat map illustration of log2-based changes in the intersection subset of genotype-independent gene regulation upon 7 days C. *rodentium* infection. Top 10 up- and down-regulated genes are shown together with significantly enriched pathways for ***c)***, ***d)*** wild-type mice and ***e)***, f***)*** N*lrp12*$^{-/-}$ mice on day 7 post-infection. Pathways framed in red are significantly enriched by up-regulated gene subsets, while those in blue are significantly enriched by down-regulated gene subsets.

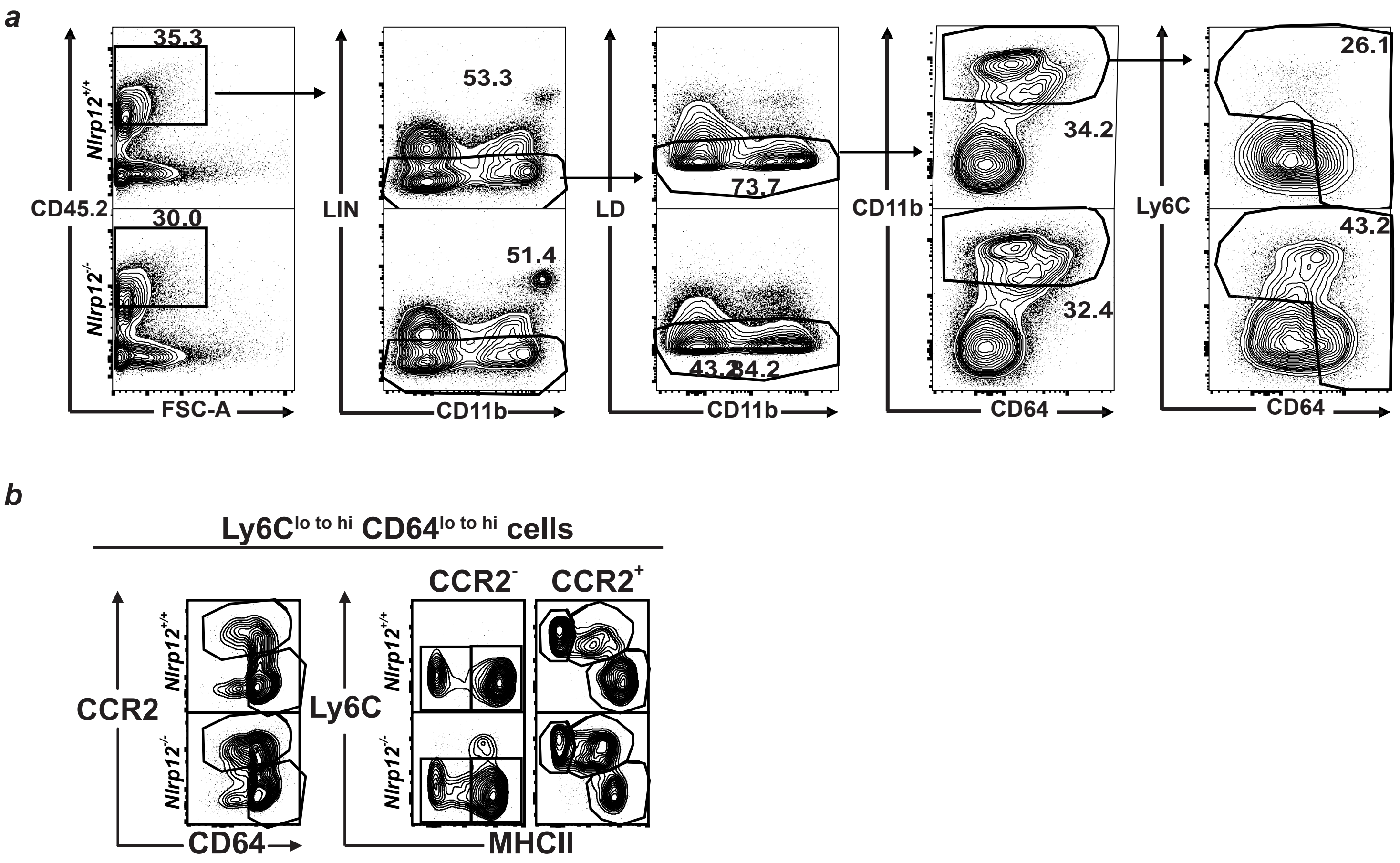

Supplementary Fig. 19. Gating strategy of the cytofluorometry analysis of cell suspensions from the colonic *lamina propria* of infected wild-type and *Nlrp12*-deficient mice. *a)* Gating strategy of the cytofluorometry analysis represented in panel ***b)***. ***b)***. Representative expression of Ly6C and MHCII expression amongst total live fraction of CCR2 and/or CD64-expressing colonic CD11b^{+} cells from *Nlrp12*$^{-/-}$ and wild-type mice.

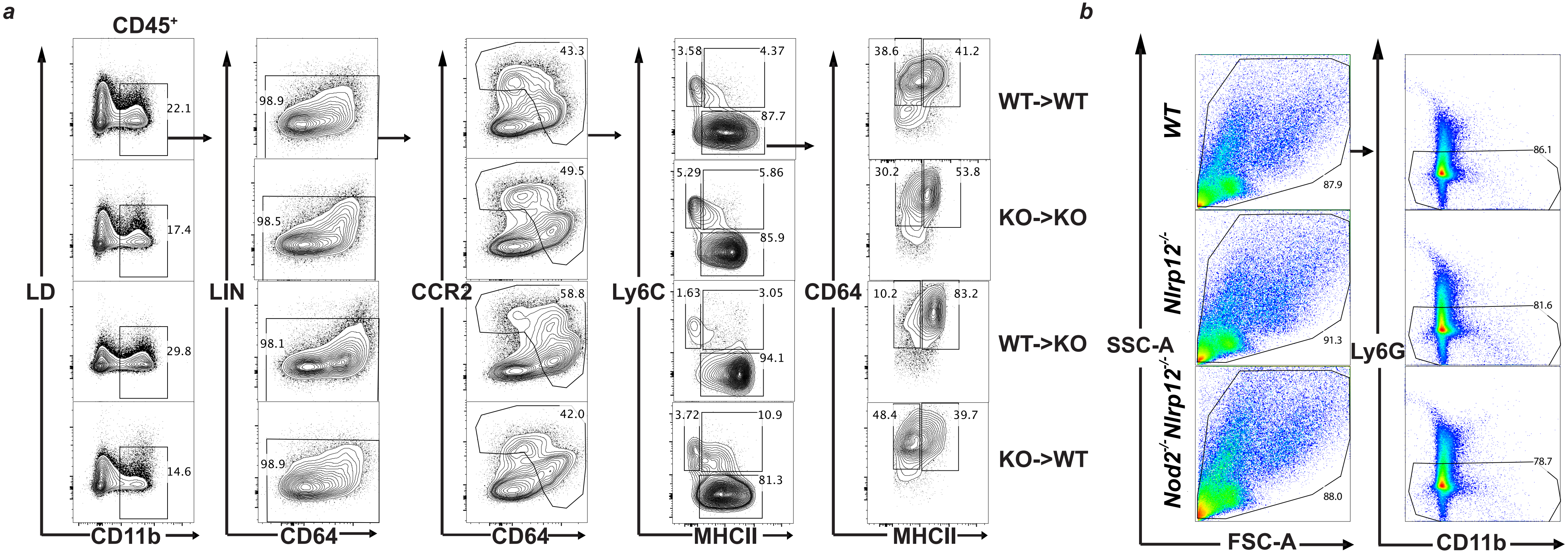

Supplementary Fig. 20. Gating strategy of the cytofluorometry analysis. Cell suspensions from the colonic lamina propria used for panel h of Figure 5 ***(a)***, and panel k of Figure 6 ***(b)***.

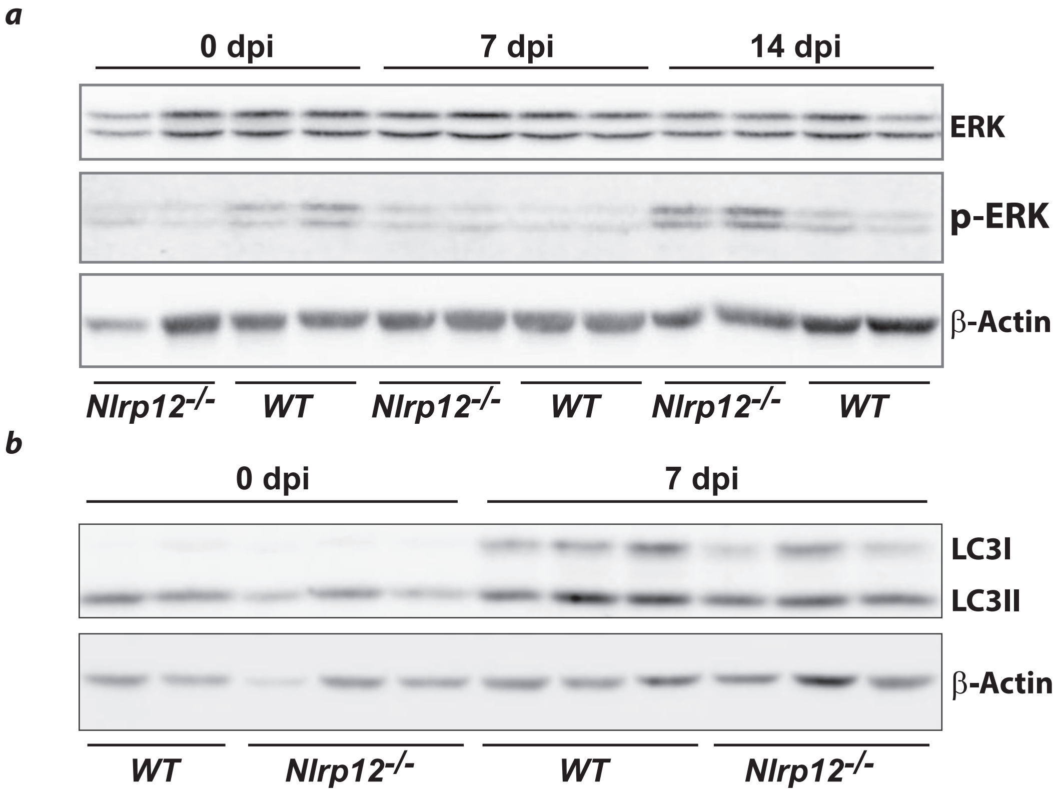

Supplementary Fig. 21. Activation of ERK pathway and lipidation of LC3 in the colon of infected mice. Western blot analysis was performed by using samples from distal colon of *Nlrp12*$^{-/-}$ and control mice on day 0, day 7, and day 14 post *C. rodentium* infection, respectively. **(a)** Activation of ERK pathway was analysed by using antibodies against ERK and p-ERK, respectively. **(b)** Activation of autophagy was analysed by using anti-LC3 antibody. β-Actin was used as loading control.

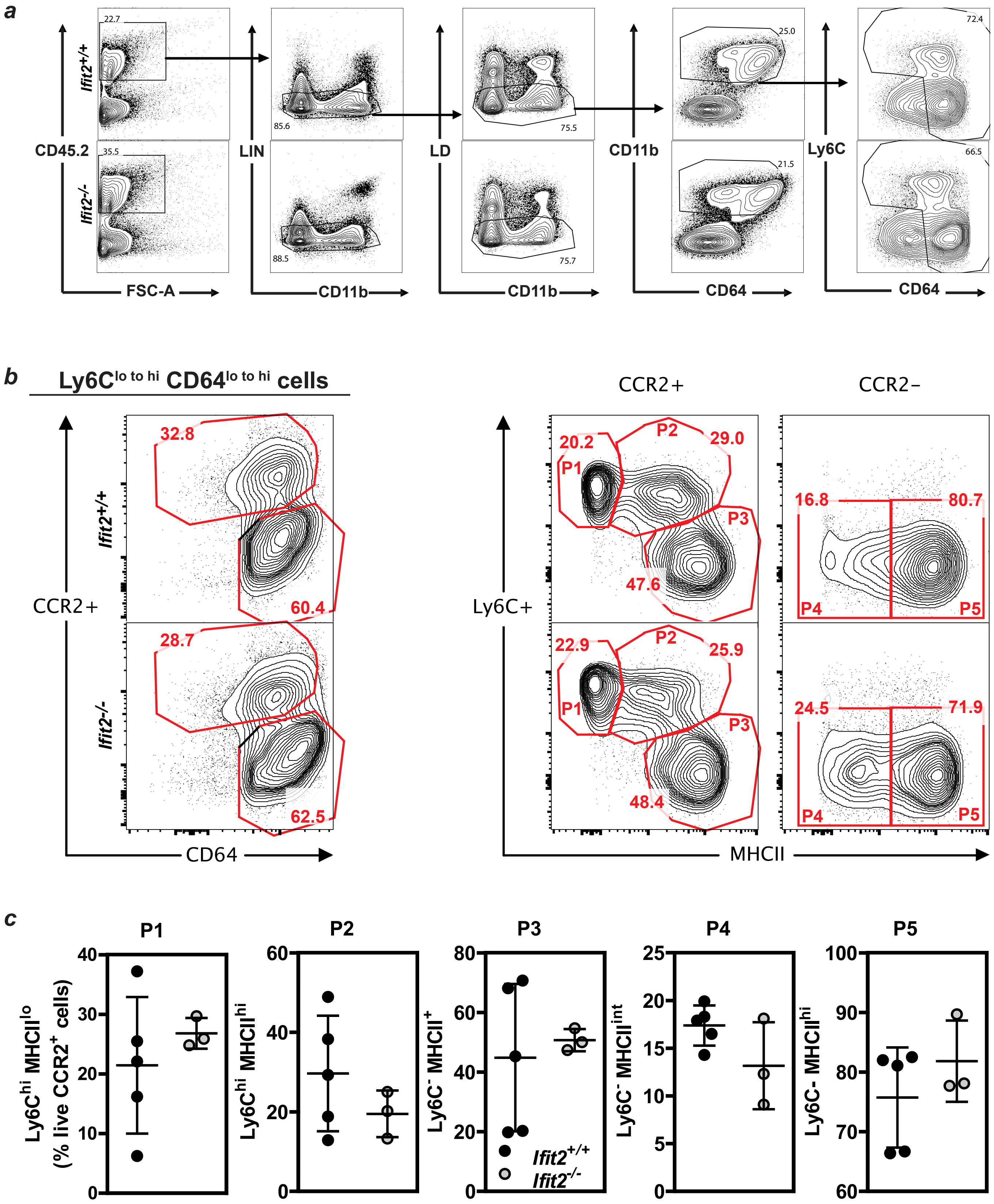

Supplementary Fig. 22. Monocyte-waterfall shaped distribution within the colon *lamina propria* of *Ifit2*-deficient mice in response to *Citrobacter rodentium*. *a)* Gating strategy of the cytofluorometry analysis. ***b)*** Representative expression of Ly6C and MHCII expression amongst total live fraction of CCR2 and/or CD64-expressing colonic CD11b^{+} cells from *Ifit2*$^{-/-}$ and wild-type mice. ***c)*** Mean proportion of Ly6C^{hi}MHCII^{lo}, Ly6C^{hi}MHCII^{+} and Ly6C^{-}MHCII^{+} subsets as a percentage of live CCR2-expressing colonic CD11b^{+} cells from the lamina propria of wild-type and mutant mice. Statistical significance was assessed by non-parametric Mann-Whitney test.
