# Supplementary material for: Proteasomal degradation of NOD2 by NLRP12 in monocytes promotes bacterial tolerance and colonization by enteropathogens
Source: Nat Commun. 2018 Dec 17;9:5338. doi: 10.1038/s41467-018-07750-5 (PMC6297353; doi:10.1038/s41467-018-07750-5)
Supplement: Supplementary file 2 — Description of Additional Supplementary Files [file 41467_2018_7750_MOESM2_ESM.docx]

**Description of Additional Supplementary Files**

File Name: Supplementary Data 1

Description: Gene expression profiles of total tissue derived from non-treated and *C. rodentium*-infected Nalp12 -/- mice (Mut) and wildtype controls (WT).
