# Supplementary material for: Proteasomal degradation of NOD2 by NLRP12 in monocytes promotes bacterial tolerance and colonization by enteropathogens
Source: Nat Commun. 2018 Dec 17;9:5338. doi: 10.1038/s41467-018-07750-5 (PMC6297353; doi:10.1038/s41467-018-07750-5)
Supplement: Supplementary file 5 — Reporting Summary [file 41467_2018_7750_MOESM5_ESM.pdf]

nature research

Corresponding author(s): NCOMMS-16-29527D

Reporting Summary

Nature Research wishes to improve the reproducibility of the work that we publish. This form provides structure for consistency and transparency in reporting. For further information on Nature Research policies, see Authors & Referees and the Editorial Policy Checklist.

Statistical parameters

When statistical analyses are reported, confirm that the following items are present in the relevant location (e.g. figure legend, table legend, main text, or Methods section).

n/a	Confirmed	
☐	☒	The exact sample size (*n*) for each experimental group/condition, given as a discrete number and unit of measurement
☐	☒	An indication of whether measurements were taken from distinct samples or whether the same sample was measured repeatedly
☐	☒	The statistical test(s) used AND whether they are one- or two-sided<br>*Only common tests should be described solely by name; describe more complex techniques in the Methods section.*
☒	☐	A description of all covariates tested
☐	☒	A description of any assumptions or corrections, such as tests of normality and adjustment for multiple comparisons
☐	☒	A full description of the statistics including central tendency (e.g. means) or other basic estimates (e.g. regression coefficient) AND variation (e.g. standard deviation) or associated estimates of uncertainty (e.g. confidence intervals)
☒	☐	For null hypothesis testing, the test statistic (e.g. *F*, *t*, *r*) with confidence intervals, effect sizes, degrees of freedom and *P* value noted<br>*Give P values as exact values whenever suitable.*
☒	☐	For Bayesian analysis, information on the choice of priors and Markov chain Monte Carlo settings
☒	☐	For hierarchical and complex designs, identification of the appropriate level for tests and full reporting of outcomes
☒	☐	Estimates of effect sizes (e.g. Cohen's *d*, Pearson's *r*), indicating how they were calculated
☐	☒	Clearly defined error bars<br>*State explicitly what error bars represent (e.g. SD, SE, CI)*

Our web collection on statistics for biologists may be useful.

Software and code

Policy information about availability of computer code

Data collection	*Provide a description of all commercial, open source and custom code used to collect the data in this study, specifying the version used OR state that no software was used.*
Data analysis	Graphpad software

For manuscripts utilizing custom algorithms or software that are central to the research but not yet described in published literature, software must be made available to editors/reviewers upon request. We strongly encourage code deposition in a community repository (e.g. GitHub). See the Nature Research guidelines for submitting code & software for further information.

Data

Policy information about availability of data

All manuscripts must include a data availability statement. This statement should provide the following information, where applicable:

- Accession codes, unique identifiers, or web links for publicly available datasets
- A list of figures that have associated raw data
- A description of any restrictions on data availability

The microarray data have been deposited under GEO accession number GSE59940.

Field-specific reporting

Please select the best fit for your research. If you are not sure, read the appropriate sections before making your selection.

☒ Life sciences ☐ Behavioural & social sciences ☐ Ecological, evolutionary & environmental sciences

For a reference copy of the document with all sections, see nature.com/authors/policies/ReportingSummary-flat.pdf

Life sciences study design

All studies must disclose on these points even when the disclosure is negative.

Sample size	Sample-size calculation was not determined but the mice experiments were repeated at least twice.
Data exclusions	Data exclusions on mice experiments was made based on Grubb's test.
Replication	All attempts at replication were succesful
Randomization	Allocation was random
Blinding	The investigators were blinded

Reporting for specific materials, systems and methods

Materials & experimental systems

n/a	Involved in the study	
☐	☒	Unique biological materials
☐	☒	Antibodies
☐	☒	Eukaryotic cell lines
☒	☐	Palaeontology
☐	☒	Animals and other organisms
☐	☒	Human research participants

Methods

n/a	Involved in the study	
☒	☐	ChIP-seq
☐	☒	Flow cytometry
☒	☐	MRI-based neuroimaging

Unique biological materials

Policy information about availability of materials

Obtaining unique materials	Patient's cells were unique.

Antibodies

Antibodies used

anti-MYC mouse antibody (9E10, 1:1000, Roche, Cat. No. M4439)
anti c-MYC rabbit antibody (1:1000, Santa Cruz, sc-789)
anti-FLAG (M2) mouse antibody (1:2000, Stratagene, #200471)
anti-beta actin antibody (1:10000, Santa Cruz, sc-47778)
anti-NLRP12 monoclonal antibody (Genetex Inc., USA, 1:1000, Cat No. GTX81551)
Anti-NOD2 (2D9) monoclonal antibody (Santa Cruz Biotechnology Inc., USA, 1:250, CatNo.sc-56168)
Anti-Myc antibody (Cell Signalling Technology, USA, 1:2500, Cat No.2272)
Anti-HSP90 (F8) monoclonal antibody (Santa Cruz Biotechnology Inc., USA, 1:1500, Cat No. sc-13119)
Anti-pUb-48 monoclonal antibody (Merck, Millipore, USA, 1:2500, Cat No. 05-1307)
Anti-alpha Actin antibody (Sigma Aldrich, UK, 1:2000, Cat No. A5060)
Anti-alpha Tubulin antibody (Sigma Aldrich, UK, 1:2000, Cat No. T9026)

Validation

Validation statements on the manufacturer's website.

Eukaryotic cell lines

Policy information about cell lines

Cell line source(s)	The THP-1 ,a monocytic cell line derived from an acute monocytic leukemia patient, was obtained from the ATCC® collection culture (ATCC®,USA)
Authentication	The authentication procedure was performed by ATCC
Mycoplasma contamination	Not contaminated
Commonly misidentified lines (See ICLAC register)	Not applicable

Animals and other organisms

Policy information about studies involving animals; ARRIVE guidelines recommended for reporting animal research

Laboratory animals	Mus musculus, male and female
Wild animals	Not applicable
Field-collected samples	Not applicable

Human research participants

Policy information about studies involving human research participants

Population characteristics	The patients were initially described in Proc Natl Acad Sci U S A. 2008 Feb 5;105(5):1614-9. doi: 10.1073/pnas.0708616105
Recruitment	The patients were included during a follow-up visit at the clinic.

Flow Cytometry

Plots

Confirm that:

☒ The axis labels state the marker and fluorochrome used (e.g. CD4-FITC).

☒ The axis scales are clearly visible. Include numbers along axes only for bottom left plot of group (a 'group' is an analysis of identical markers).

☒ All plots are contour plots with outliers or pseudocolor plots.

☒ A numerical value for number of cells or percentage (with statistics) is provided.

Methodology

Sample preparation	*Describe the sample preparation, detailing the biological source of the cells and any tissue processing steps used.*
Instrument	FACS Canto II (BD biosciences) and FACS LSRFortessa system (BD Biosciences)
Software	FlowJo software (Tree Star)
Cell population abundance	Not applicable
Gating strategy	The gating strategy is described in supplementary figures 14, 16 and 17

☒ Tick this box to confirm that a figure exemplifying the gating strategy is provided in the Supplementary Information.
